# London as a Medical Centre

**Published:** 1906-09-01

**Authors:** 


### The Hospital

A Journal of

The Medical Sciences and Hospital Administration.

Vol. XL.—No. 1,041. SATURDAY, SEPTEMBER 1, 1906.

### EDUCATIONAL NUMBER.

### London as a Medical Centre.

### Industry and Capacity.

The selection of a medical school for a youth intent on entering the profession of medicine is often a matter of considerable anxiety for those with whom lies the responsibility of the final decision. And it cannot be denied that the question is one of some importance. In all probability, however, the choice carries less serious issues than it seems to do to those burdened with the necessity of speaking the fateful word. For whatever school be ultimately selected, the results of the educational process will depend fully as much on the candidate himself as upon the facilities and opportunities placed at his disposal. Attention, industry, capacity, perseverance, will make even limited chances yield the material for thorough and satisfactory self-discipline, whilst carelessness or laziness will permit a perfect wealth of opportunity to pass away without use or purpose. Further, at the present day, intercommunication and the stress of competition have brought all medical schools of any considerable reputation and position to much the same level of efficiency. One part of the curriculum may be better organised in one school than others, but taken throughout, the general average is much the same in all.

### The Influence of Personality.

There is, however, one factor which, could it only be known and measured, would be of prime importance in making choice of a school for an aspirant for medical qualifications. This is the influence of personality. A really great teacher and worker, full of zeal and enthusiasm for his work, and with the capacity and desire to influence and guide the minds of youth, is an educational force the value of which is beyond telling. To secure this chance for any lad is to give him an educational opportunity far in advance of anything which can be obtained from perfection in such mechanical arrangements as laboratory opportunities and a large and well-equipped hospital. In medical education, in short, as indeed in all education, it is "the spirit that quickeneth; the flesh profiteth nothing." All this is the more to be remembered seeing that medical training is not the preparation for an art where similar experiences continually repeat themselves. The object of the teacher is not, or is not mainly, to make his pupils know so many facts in biological science and so many of the conditions and evidences of disease. Rather the ambition is to train the student in an appreciation of principles that will guide and direct him among the many novel situations which are sure to present themselves in the course of his future career. Hence out of the comparatively limited opportunities of a small school and hospital a really great teacher will provide an atmosphere (to use a word familiar in recent educational discussions) the absence of which will leave the most pretentious establishment and institution cold, barren, and lifeless. All this, it seems reasonable and proper to say to the guardians and advisers of those about to enter on the search for a medical career. No general scholastic prestige or reputation will atone for the absence of imagination and teaching ability among the present staff of even a considerable school, and without personal resolve and endeavour on the part of the student himself, the best of opportunities will be offered in vain.

### London as a Centre.

Having then said so much with a view to place the choice of a medical school in its proper perspective, it may now be well to consider some practical points which must be weighed within the limits assigned by the foregoing remarks. The question of geography or proximity, as involving the consideration of convenience and expense, must in many cases be final, and, as already explained, given the right student and the right teacher, this need involve no regret. But with a completely free choice it is almost inevitable that the thoughts both of the candidate and his guardians should be turned to London. And in one respect, at all events, no one can deny that London as a centre for medical education boasts opportunities with which no other city can successfully compete. Nowhere else is there to be found such a wealth and variety of disease. This is contributed not only by its own enormous population but also by numerous cases which are attracted from all parts of the country by the general fame and reputation of the metropolis. More particularly is this true, perhaps, of those so-called special forms of disease which lead to the cultivation of special branches of practice. The metropolitan hospitals concerned with, for example, diseases of the eye, ear, throat, skin, and nervous system, are many of them of world-wide reputation and afford the material necessary for clinical study in the very highest quantity and value. From this it necessarily follows that London possesses the largest number of physicians and surgeons having a special knowledge of these diseases and specially able to give authoritative instruction regarding them.

The Student's Opportunities.

In comment upon what has just been said it may be objected that all this has much more to do with post-graduation work than with the training of the undergraduate. Such a remark, though appropriate to some extent, is however only true in part. The medical student of the present day, with his five years' curriculum, is compelled to include some branches of special work within his period of study, and a really industrious student can readily manage to undertake more than is technically necessary. Hence opportunities for this class of work are not to be despised, even in pre-graduation days. Further, what is true in particular measure of special practice also applies largely to the more ordinary teaching of medicine and surgery. Some of the metropolitan hospitals possess in these respects clinical opportunities that cannot be surpassed, and this refers not only to the number and variety of the cases but also to the ability and reputation of the teachers. Among these are men famous not only for their skill as physicians and surgeons, but also for their ability to inspire their pupils with devotion to the highest ideals and to the loftiest conceptions of professional duty and responsibility. To the value of these latter influences reference has already been made, and their range and importance in medical practice can hardly be exaggerated.

Excellent Organisation of the Out-patient Department.

If one particular feature of the clinical schools in London may be emphasised it is the value and excellent organisation of many of the out-patient departments. Whatever economic criticism may be passed upon these, they may without question be made valuable training schools for medical practice. Here are to be met the ordinary ailments which afflict humanity, rather than the curious and unusual conditions which, however great their pathological interest, the student is hardly likely to see more than once in a lifetime. The out-patient department, in short, when rightly used, can be made the best practical preparation for the claims of general practice. And, in at least some of the London schools, the opportunities and organisation are most excellent.

General Value of London.

Besides all these specific values there is in addition something to be said in favour of the general value of London as an educational centre. Into this great city are collected influences and emotions gathered from all parts of the globe. The claims of the village pump, though they exist, are subordinated to and concealed by the stirrings of larger and broader issues. The air is quick and tense with the movement of many minds and of influences well calculated to widen the horizon and stir the ambitions of courageous and generous youth. Temptations in almost corresponding measure go along with these great opportunities, but even from these may be won the capacity for self-control " and all that makes a man." Altogther it is not a light thing for a resolute and high-minded youth to enjoy the opportunity to spend the formative years of his life amidst the stimulating forces which stir and vibrate in the centre and heart of the British Empire. So far to the praise and advantage of London.

Spots on the Sun.

There are, however, spots on the sun, and metropolitan medical education is not without its defects. These exist more particularly in the earlier or so-called scientific subjects of the medical curriculum. With each of the larger hospitals endeavouring to develop an independent and complete medical school, it is impossible in every instance to raise funds adequate to equip proper laboratories or to pay efficient teachers. Hence work in these departments often falls into the hands of those of junior experience who use their immediate appointments as a vaulting-horse to larger ambitions. The question is one mainly of ways and means and it will never be solved until the various schools recognise that London must remain, so far at least as the earlier part of the curriculum is concerned, hopelessly behind the Scottish and provincial universities until the present system of multiplied teachers in the earlier subjects is abandoned. This conviction is already showing signs of activity and, probably before many years, will have penetrated even the most parochial of the metropolitan schools. With one, or possibly two, well-endowed colleges including professorships with adequate salaries, and modern laboratories dealing with the subjects that form the scientific basis of medicine, the clinical opportunities of London would receive their due association, and in consort, the combination would form a school for medical training and research worthy of the metropolitan city. At present, the absence of such an arrangement is a distinct disadvantage, yet in spite of this, and for reasons already given, London presents attractions and opportunities which may well appeal to the earnest and ambitious student.

